# Why and how power disparity harm team performance

**DOI:** 10.3389/fpsyg.2025.1702275

**Published:** 2025-12-10

**Authors:** Shiwen Luo, Fumitaka Furuoka, Xuelian Zuo

**Affiliations:** 1 Zhejiang Financial College, Hangzhou, China; 2 Universiti Malaya, Federal Territory of Kuala Lumpur, Kuala Lumpur, Malaysia; 3 Zhejiang Yuexiu University, Shaoxing, China

**Keywords:** power disparity, power congruence, task conflict, relationship conflict, team performance

## Abstract

**Introduction:**

Power disparity, defined as the unequal control over valuable resources within teams, has been shown to negatively impact overall team performance, though the mediating mechanisms and boundary conditions of this relationship remain inadequately explored. Grounded in power conflict theory, this study develops a conceptual model that incorporates team conflict—comprising both relationship conflict and task conflict—as a mediator, and power congruence as a moderator, to systematically explain why and how power disparity influences team performance.

**Methods:**

Survey data were collected from 62 teams. The proposed hypotheses concerning the mediating and moderating effects were tested through a series of statistical analyses, including reliability and validity tests, confirmatory factor analysis, and hierarchical regression.

**Results:**

Power disparity was found to negatively affect team performance through increased relationship conflict, while also positively influencing team performance via task conflict. However, the negative indirect effect through relationship conflict was significantly stronger than the positive effect through task conflict. Furthermore, power congruence moderated these mediating pathways: it attenuated the negative effect of relationship conflict and strengthened the positive effect of task conflict. These findings reveal a dual pathway through which power disparity affects team performance, with the overall effect being negative due to the stronger detrimental role of relationship conflict. The moderating role of power congruence helps mitigate harmful conflict and promotes beneficial debate, offering theoretical contributions to power conflict theory and practical implications for designing more effective team power structures.

## 1 Introduction

As an asymmetric control over valuable resources in social relationships, power is ubiquitous and universal in social interactions (Magee and Galinsky, 2008; Korman et al., 2022). Power has been proven to provide individuals with a series of benefits, such as the enhancement of implementation capacity (Zheng et al., 2023), the improvement of life satisfaction (Anderson et al., 2012), the realization of goal vision and the extension of life expectancy (Greer et al., 2017). However, in an interdependent team environment, the effects of power are more complex; while it may enhance team performance, it can also potentially harm it (Brief and Smith-Crowe, 2016; Greer and Chu, 2020). For example, team leaders, through strong decision-making power, can effectively and quickly drive project progress. However, at the same time, this highly concentrated power may also lead to conflicts among team members, affecting collaboration and team innovation. In research on team power, scholars have attempted to conceptualize power from different perspectives. To date, two main conceptualizations of team power have emerged: power level and power disparity (Greer et al., 2017; Luo and Tong, 2024). Power level and power disparity are two distinct aspects of team power structure. Power level (Greer and van Kleef, 2010) describes the *average* extent of power resources (e.g., control over resources and decision-making authority) within a team, typically categorized as high or low. Conversely, power disparity (Tarakci et al., 2016, p. 416) reflects the distribution or concentration of these resources among members. It ranges from high (resources concentrated in one member) to low (resources evenly distributed). Due to the widespread existence of power imbalance, research has focused more on power disparity rather than power level, and has found that power disparity is closely related to various team outcomes (Greer et al., 2017; Xie et al., 2020; Cheng and Sandfort, 2023).

Academic discourse on power disparity features two main opposing views. Scholars of power functionalism highlight its positive effects, arguing that it establishes a clear collaborative order by defining decision-making authority and role expectations. This order helps reduce conflicts over ambiguous responsibilities, facilitates internal coordination and division of labor, and motivates voluntary cooperation, thereby effectively enhancing team performance (Greer et al., 2017; Halevy et al., 2011; Zhu et al., 2019; Gobel and Miyamoto, 2023). Conversely, scholars of power conflict theory focus on its negative effects. They point out that power disparity may trigger perceptions of unfairness in resource and status distribution, setting off a chain of negative mechanisms: cognitively, members tend to attribute the disparity to procedural injustice, reinforcing relative deprivation (Smith et al., 2012); emotionally, unfairness fosters jealousy and resentment, undermining emotional bonds (Greer and van Kleef, 2010; Sinha et al., 2021); behaviorally, these reactions lead to non-cooperative competition and power struggles (Bunderson et al., 2016; Greer et al., 2018). The cumulative effect erodes trust, diverts attention from tasks, and ultimately damages team performance (Lam et al., 2023; Ling and Luo, 2024). Scholars have continuously explored these two perspectives. However, based on meta-analytic results, the main effect of power disparity is negative, and the power functionalism perspective is largely unsupported (Greer et al., 2018; De Wit et al., 2012; Greer et al., 2017; Ling and Luo, 2024). Furthermore, recent research has deepened the explanatory dimensions of power conflict theory. For instance, Rabenu's (2021) “team pack” metaphor and LMX differentiation theory show that the “insider-outsider” division solidifies perceptions of power imbalance and exacerbates cooperation dilemmas. Separately, Zverling (2019), from a perspective of power insecurity, reveals that when individuals in power feel their authority is threatened, they are more inclined to adopt domineering behaviors. This response aggravates conflicts, thereby creating a vicious cycle. This suggests that the negative effects of power disparity often outweigh its potential positive effects. Therefore, further research on power conflict theory, particularly on the conditions under which and the reasons why power disparity may harm team performance, is of great importance. Understanding this mechanism not only helps reveal the negative effects of power disparity but also provides a theoretical basis for organizations to design rational power structures and implement effective interventions to prevent the negative consequences of overly centralized power structures.

In recent years, scholars have increasingly recognized that the effect of power disparity on team performance is not direct but operates through complex mediating mechanisms and boundary conditions (Greer et al., 2018; Luo and Tong, 2024). A key mediator from the perspective of power conflict theory is team conflict, which helps explain the negative consequences of such disparity (Xie et al., 2020; Greer et al., 2017; Zhu et al., 2019). Significant power disparity often leads to an asymmetric control over valuable resources (Magee and Smith, 2013), creating a divide between members with abundant resources and those facing scarcity. In resource-scarce and competitive environments, this asymmetry can easily trigger competition and confrontation (Greer and van Kleef, 2010), resulting in team polarization and power struggles (Keltner et al., 2008). This dynamic erodes interpersonal trust and intensifies relational tensions (Bunderson et al., 2016), thereby raising the overall level of team conflict. These conflicts are further exacerbated by the divergent motivations and interests between lower-power members, who strive for more resources and authority, and higher-power members, who seek to preserve their advantages (Greer et al., 2017). Although substantial research confirms the generally detrimental effect of team conflict on performance (Jehn et al., 2008), existing studies have predominantly treated it as a unidimensional construct. This approach overlooks the diversity of conflict types and their potentially differentiated impacts on team outcomes (Zhu et al., 2019; Foncubierta-Rodríguez et al., 2022; Simons and Peterson, 2000). For example, relationship conflict typically undermines trust and cooperation among team members, reduces team cohesion and collaboration efficiency (Alves et al., 2023), ultimately leading to a negative effect on team performance. In contrast, task conflict can foster the exchange of diverse perspectives among team members, stimulating creativity and varied thinking approaches, thereby enhancing decision-making quality and efficiency (Jia et al., 2024), which in turn promotes team performance. If different types of conflict are not distinguished, the positive effects of task conflict may be overlooked, particularly in complex tasks that require innovation and diverse thinking. This could lead to an overemphasis on the negative aspects of conflict, resulting in the misleading conclusion that “conflict is harmful”. Moreover, this neglect may prevent a clear understanding of how power disparity influences team performance through different types of conflict, thereby rendering the explanation of the mechanism of power disparity incomplete or biased, or leading to inaccurate evaluations of overall team performance. To address this, scholars have suggested distinguishing between types of conflict and exploring the different mediating roles that conflict types play in the relationship between power disparity and team performance (Zhu et al., 2019). Therefore, drawing on classic literature, this study classifies conflict into relationship conflict and task conflict (Jehn, 1995), and investigates how power disparity affects team performance through team conflict along these two different paths, thereby revealing the underlying mechanism of how power disparity influences team performance through team conflict under power conflict theory.

According to contingency theory, the effects of power disparity are moderated by a range of factors. Existing research mainly focuses on factors such as task type, leadership characteristics, and team structure (Anderson and Willer, 2014; Greer, 2014; Bunderson et al., 2016; Ronay et al., 2012; Xie et al., 2020; Zhu et al., 2019; Yu and Greer, 2023); however, the effect of power itself has rarely been considered. Power itself, as a potential moderator, has not been fully explored in terms of its role in team power structures (Greer, 2014), and such research is considered an important direction for future academic exploration. Specifically, the mechanisms through which power itself influences team performance mainly include two aspects: first, different dimensions of the power structure, such as power level and power diversity (Groysberg et al., 2011); second, the psychological mechanisms through which power exerts its effects, such as power cognition (Greer, 2014). Although there has been relatively in-depth exploration of power structures, research on the psychological mechanisms through which power exerts its effects, particularly on power cognition, remains insufficient and is considered an important direction for future studies. Power cognition consists of two key constructs: power congruence and power legitimacy (Greer, 2014). Specifically, power congruence refers to the degree of cognitive agreement among team members regarding their own and others' relative positions within the team (Polzer et al., 2002; Greer et al., 2011), and this congruence significantly influences the effectiveness of the team power structure (Greer et al., 2017). Research has shown that when team members reach a consensus on the power structure, they are able to clearly understand their position within the team, thereby reducing anxiety, feelings of threat, and confrontational behavior during interactions (Martorana et al., 2005). This cognitive congruence can reduce conflicts within the team, thereby promoting overall collaboration and performance. In contrast, if there is a cognitive divergence regarding the power structure among team members, some may overestimate their position in the team, which could not only lead to rejection and punishment by others but also intensify conflicts within the team (Anderson et al., 2008; Greer, 2014), ultimately harming team performance. Therefore, power congruence may improve the negative effects of power disparity by reducing internal team conflicts. Based on this, this study will attempt to explore the interaction between power congruence and power disparity from the psychological mechanism of power itself, analyzing how it influences team performance through its effect on internal conflicts (including relationship and task conflicts). This research perspective not only helps deepen the understanding of the complexity of the effects of power disparity but also provides new insights for future studies in organizational behavior, team management, and power structure optimization.

## 2 Theoretical analysis

### 2.1 The mediating role of team conflict

Power disparity is an important research concept associated with power in the context of group interaction, and it has also received a large amount of attention in the research in recent years. Meta analyses show that the overall effect of power disparity on team performance is negative, and team conflict is the key process explanatory variable (Greer and Chu, 2020; Greer et al., 2018). Conflict refers to a situation of inconsistency among group members' views and disharmony in interpersonal relationships, and it mainly includes relationship conflict and task conflict (Jehn, 1995). The power conflict theory claims that power disparity leads to negative emotions such as perceptions of unfairness, worsens the interpersonal relationships, and causes relationship conflicts (Greer et al., 2017). Power disparity also leads to divisions and power struggles within the team, thus triggering relationship conflicts among group members (Zhu et al., 2019; Hekkala et al., 2022). Bunderson et al. (2016) investigated 75 teams working in multiple industries to confirm that power disparity affects relationship conflict positively. Additionally, power disparity is often accompanied by an uneven allocation of resources (Anderson and Willer, 2014), where high-power members may control more resources. This can lead low-power members to feel unfairly treated, prompting them to question the resource allocation (Greer et al., 2018). When team members have divergent views on resource distribution, task conflict is likely to arise (Ling and Luo, 2024). At the same time, when team members perceive power differences, they may develop differing views on task goals, priorities, and execution methods (Greer and Chu, 2020). High-power members may dominate decision-making and push their own task goals, while low-power members may feel ignored, leading to disagreements and goal conflicts, which further intensify task conflict. Therefore, power disparity can trigger both relationship and task conflicts within the team. Based on this, the following hypotheses are proposed.

**H**_**1**_**:** *Relationship conflict plays a mediating role in the relationship between power disparity and team performance. In other words, power disparity harms team performance by triggering relationship conflict*.

**H**_**2**_**:** *Task conflict plays a mediating role in the relationship between power disparity and team performance. In other words, power disparity enhances team performance by triggering task conflict*.

### 2.2 The moderating role of power congruence

As one of the psychological mechanisms through which power exerts its effects, power congruence is an important component of power cognition. It is defined as the cognitive congruence of team members regarding their relative hierarchical order or position, that is, the degree to which team members align on their positions within the team (Greer et al., 2017). Research has shown that power congruence plays a crucial role in the effectiveness of team power structures and can alleviate team conflicts arising from power disparity (Greer et al., 2011; Greer, 2014). Specifically, power disparity often comes with some members controlling resources, which can trigger feelings of threat or unfairness among low-power members, leading to relationship conflict (Greer et al., 2017). However, in teams with high power congruence, members‘ cognition of the power structure and resource distribution tend to align, and this congruence eliminates low-power members' doubts and fears of unfair treatment. Power congruence not only fosters members‘ identification with the power structure and resource allocation, but also effectively reduces negative emotions, such as anger, dissatisfaction, and jealousy, which are often the root causes of relationship conflict (Greer, 2014). When team members can identify with the team's power distribution, they are more likely to handle issues arising from power disparity rationally, avoiding emotional conflicts. This emotional relief helps reduce interpersonal conflicts, thus mitigating the positive effect of power disparity on relationship conflict. Furthermore, power congruence provides higher stability and predictability, making communication among team members more efficient. When members understand each other's roles and power positions, communication within the team flows more smoothly, and conflicts tend to focus on the tasks themselves rather than interpersonal conflicts (Butchibabu et al., 2016). In an environment of high power congruence, team members are more likely to identify with and accept each other's task goals, thereby enhancing teamwork. Despite the presence of power disparity, members' recognition and understanding of task goals allow task conflict to become a constructive force rather than a destructive one. This efficient cooperation mechanism promotes the positive effect of task conflict and helps team members form more efficient collaboration in task execution (Greer and van Kleef, 2010). In contrast, when team members have differing views of the power structure, i.e., low power congruence, members may be unclear about their position within the team, which can lead them to overstep their role boundaries, provoking threats and retaliation from other members, thus escalating relationship conflict (Owens and Sutton, 2014; Anderson et al., 2008). Low power congruence may also disrupt the communication climate within the team, hindering the effective sharing of information among team members, thus lowering the level of task conflict. Therefore, power congruence plays an important moderator in the relationship between power disparity and team conflict. Based on this, the following hypotheses are proposed.

H_3_: *Power congruence plays a moderating role in the relationship between power disparity and relationship conflict; that is, power congruence can mitigate the positive relationship between power disparity and relationship conflict*.

H_4_: *Power congruence plays a moderating role in the relationship between power disparity and task conflict; that is, power congruence can enhance the positive relationship between power disparity and task conflict*.

Based on the above analyses and theoretical assumptions, this study further posits that power congruence can moderates the mediating effect of team conflict in the relationship between power disparity and team performance. Specifically, under high power congruence, the positive relationship between power disparity and relationship conflict is alleviated, while the positive relationship between power disparity and task conflict is strengthened, ultimately leading to an improvement in team performance. Therefore, this paper proposes the following integrated hypotheses:

H_5_: *Power congruence moderates the mediating effect of relationship conflict in the relationship between power disparity and team performance; that is, the higher power congruence is, the weaker the negative effect of power disparity on team performance via relationship conflict*.

H_6_: *Power congruence moderates the mediating effect of task conflict in the relationship between power disparity and team performance; that is, the higher power congruence is, the stronger the positive effect of power disparity on team performance via task conflict*.

**Figure 1:**
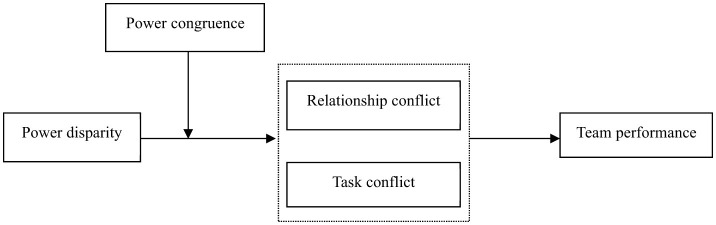
Theoretical model.

The theoretical framework of this study is shown in Figure 1.

## 3 Method

### 3.1 Participants and procedure

The data referenced by this study were collected using the questionnaire survey method. The respondents were drawn from 74 teams associated with 18 enterprises in China, including the departments of production technology, financial accounting, human resource management, marketing promotion, and after-sales service in the textile. To mitigate common method bias, this study employed a two-wave time-lagged survey design with a 3-month interval. This approach temporally separated the measurements of predictor variables (power disparity, team conflict) from the outcome variable (team performance). Such separation reduces the likelihood of respondents making artificial causal connections between the measures, thereby enhancing the discriminant validity and strengthening the causal interpretability of the findings. During the first stage, questionnaires were distributed to 415 participants from 74 teams, with the aim of measuring power disparity, power congruence, task conflict, and relationship conflict.0 389 valid questionnaires from 69 teams were retained, with an recovery rate of 93.73%. Three months later, 389 questionnaires were returned to the original team members, which mainly measured team performance. After removing invalid questionnaires with same questions, 351 valid questionnaires from 62 teams were obtained, with an recovery rate of 90.23%. An analysis of the valid questionnaires collected in the second stage revealed that males accounted for 63.53% of the total sample and females accounted for 36.47%; 45.87% of respondents had college degrees or lower levels of education, 36.47% were undergraduates, and 17.66% were postgraduates; 29.06% of the employees had worked on their team for 3 years or more, 44.16% had worked on their team for 1–3 years and 26.78% had worked on their team for less than 1 year; and the average team size was 5.66 people, with teams featuring more than 10 people accounting for 6.45% of the total, teams featuring 5–10 people accounting for 48.39%, and teams featuring 3–5 people accounting for 45.16%.

### 3.2 Measurements

#### 3.2.1 Power disparity

In this study, the rotating questionnaire design method is used to measure power disparity. The specific approach is for each team member to evaluate the power level of other team members based on the 5-point Likert scale (1 means “almost none” and 5 means “a great deal”). The measurement item is: “ How much power do I think this colleague has in the team (for example, he or she has asymmetric control over resources and is able to ensure that the other members implement his or her own wishes)”. Subsequently, calculate the power level of a team member by averaging the evaluation scores provided by others. Next, the dispersion coefficient is calculated to reflect power disparity within the team. The larger the dispersion coefficient, the higher the degree of power disparity within the team.

#### 3.2.2 Relationship conflict

The scale draws inspiration from the research of Jehn (1995), mainly including four items, such as “how much friction is there between team members”. The Cronbach's α is 0.93.

#### 3.2.3 Task conflict

The scale draws inspiration from the research of Zhao et al. (2019), also including four items, such as “when discussing work issues, team members propose many inconsistent views or opinions”. The Cronbach's α is 0.90.

#### 3.2.4 Power congruence

This study adopts the method developed by Greer et al. (2011) to measure power congruence. First, each team member is required to evaluate the ability of everyone on the team (including himself or herself) to exert influence. The evaluation item is “how much influence each member has on the team”. Second, we calculate the differences score between self-evaluation and others' evaluation, and average the difference scores of all members to obtain the power incongruence score for each member. Furthermore, average the power incongruence score of all members and reverse code the team level incongruence score to reflect power congruence at the team level.

#### 3.2.5 Team performance

The scale draws inspiration from the research of Tjosvold et al. (2006), mainly including six items, such as “we can complete or even exceed the task”. The Cronbach's α is 0.87.

In addition, since team size, gender diversity, team diversity and team tenure have effects on team process and results (Lepine et al., 2008; Zhu et al., 2019; Ji et al., 2019; Koopmann et al., 2016), this study controls for team size, gender diversity (the Blau index is used to reflect the dispersion of the male–female ratio on the team), educational diversity (expressed in terms of the discrete coefficient) and team tenure.

## 4 Results

### 4.1 Data aggregation

Since this study is a team level related study, task conflict, relationship conflict, and team performance data are all measured by individual participants, it is necessary to aggregate the data of these variables at the team level. Aggregation conditions are usually determined based on three indicators: Rwg (consistency requirements within the group; the critical value is 0.70), ICC(1) (consistency within the group; the critical value is 0.5), and ICC(2) (consistency among groups; the critical value is 0.50). According to Table 1, the values of Rwg, ICC(1) and ICC(2) for task conflict, relationship conflict, and team performance all research the critical criteria, so aggregation can be carried out.

**Table 1:** The results of data aggregation.

**Variables**	**Rwg**	**ICC(1)**	**ICC(2)**
Relationship conflict	0.794	0.314	0.685
Task conflict	0.802	0.277	0.711
Team performance	0.834	0.309	0.623

### 4.2 Confirmatory factor analysis

To verify the discriminant validity among various factors, confirmatory factor analysis (CFA) was performed on the main variables, and the results are shown in Table 2. As shown in Table 2. From Table 2, it can be seen that compared with other sub-models, the four factor model has the better fitting accuracy: χ^2^/*df* = 1.603, RMSEA = 0.064, NNFI = 0.930, CFI = 0.930, IFI = 0.931, and is significantly better than other models, indicating good discriminant validity among variables.

**Table 2:** The results of confirmatory factor analysis.

**Models**	**χ^2^/*df***	**RMSEA**	**NNFI**	**CFI**	**IFI**
PD; RC; TC; PC; TP	1.603	0.064	0.930	0.930	0.931
PD; RC; TC; PC+TP	2.142	0.109	0.872	0.872	0.873
PD; RC; TC+PC+TP	3.722	0.175	0.832	0.833	0.833
PD; RC+TC+PC+TP	4.164	0.266	0.786	0.785	0.786
PD+RC+TC+PC+TP	6.321	0.307	0.745	0.744	0.745

*PD, power disparity; RC, relationship conflict; TC, task conflict; PC, power congruence; TP, team performance*.

### 4.3 Descriptive statistics and correlation analysis

The descriptive statistics and correlation coefficient results between variables are shown in Table 3. As shown in Table 3, power disparity is significantly positively correlated with relationship conflict (*r* = 0.227, *P* < 0.01) and significantly positively correlated with task conflict (*r* = 0.157, *P* < 0.05); task conflict is positively correlated with team performance (*r* = 0.125, *P* < 0.05); and relationship conflict is negatively correlated with team performance (*r* = −0.312, *P* < 0.01). In addition, no significant correlation was found between relationship conflict and task conflict, which suggests that it is reasonable to distinguish between relationship conflict and task conflict in team conflicts.

**Table 3:** The results of descriptive statistics and correlation analysis.

**Variables**	**M**	**SD**	**TS**	**GD**	**ED**	**TT**	**PD**	**RC**	**TC**	**PC**	**TP**
TS	5.661	1.173	—								
GD	0.247	0.062	0.093	—							
ED	0.287	0.064	0.044	0.049	—						
TT	2.611	0.605	0.043	0.006	0.039	—					
PD	0.244	0.065	−0.085	−0.061	−0.024	−0.055	—				
RC	3.613	0.512	0.020	−0.049	0.039	−0.121^*^	0.227^**^	—			
TC	2.456	0.493	−0.022	0.106	0.063	−0.006	0.157^*^	0.017	—		
PC	0.758	0.074	−0.018	0.053	0.043	−0.021	0.124^*^	0.187^**^	0.029	—	
TP	3.871	0.418	−0.009	−0.046	−0.037	−0.068	−0.173^**^	−0.312^**^	0.125^*^	0.102^*^	—

^*^P < 0.05; ^**^P < 0.01; *TS, team size; GD, gender diversity; ED, education diversity; TT, team tenure; PD, power disparity; RC, relationship conflict; TC, task conflict; PC, power congruence; TP, team performance*.

### 4.4 Hypothesis testing

#### 4.4.1 The mediating role of relationship conflict

The study used linear regression to test the relevant hypotheses (see Table 4). The results indicate that after controlling for factors such as education diversity, team size, team tenure, and gender diversity, the theoretical hypotheses are valid. The results of M_1_ show a significant negative correlation between power disparity and team performance (β = −0.151, *P* < 0.05). Meanwhile, the results of M_4_ show a significant positive correlation between power disparity and relationship conflict (β = 0.322, *P* < 0.01). In addition, the results of M_2_ show a significant negative correlation between relationship conflict and team performance (β = −0.317, *P* < 0.01), and the effect of power disparity on team performance remains significant (β = −0.131, *P* < 0.05), thus H_1_ is validated.

**Table 4:** The results of mediating effect and moderating effect analyses.

**Variables**	**TP**			**RC**			**TC**		
	**M**_1_	**M**_2_	**M**_3_	**M**_4_	**M**_5_	**M**_6_	**M**_7_	**M**_8_	**M**_9_
TS	0.032	0.031	0.037	0.005	0.012	0.014	−0.063	−0.057	−0.055
GD	−0.024	−0.022	−0.027	0.029	0.001	−0.118	0.040	0.011	0.013
ED	0.000	0.000	−0.025	0.033	0.029	0.037	0.045	0.041	0.040
TT	−0.031	−0.030	−0.027	−0.025	−0.021	−0.085	−0.050	−0.046	−0.049
PD	−0.151^*^	−0.131^*^	−0.116^*^	0.322^**^	0.224^**^	0.164^**^	0.132^*^	0.130^*^	0.144^*^
RC		−0.317^**^							
TC			0.173^**^						
PC					0.188^**^	0.285^**^		0.187^**^	0.233^**^
PD^*^PC						−0.167^**^			0.135^*^
R^2^	0.046	0.290	0.051	0.059	0.094	0.120	0.152	0.186	0.184
ΔR^2^	−0.039	0.239	−0.053	−0.025	−0.005	0.006	0.076	0.097	0.080

^*^P < 0.05; ^**^P < 0.01; *TS, team size; GD, gender diversity; ED, education diversity; TT, team tenure; PD, power disparity; RC, relationship conflict; TC, task conflict; PC, power congruence; TP, team performance*.

#### 4.4.2 The mediating role of task conflict

The results of M_7_ show that power disparity has a significant positive effect on task conflict (β = 0.132, *P* < 0.05). Meanwhile, The results of M_3_ show that task conflict has a significant effect on team performance (β = 0.173, *P* < 0.01), and the relationship between power disparity and team performance remains significant (β = −0.116, *P* < 0.05), thus H_2_ is validated.

To further validate the mediating role of team conflict, this study used the Process method and conducted 5,000 bootstrap samples to test its significance. The results indicate that, after controlling for relevant variables, the negative indirect effect of power disparity on team performance through relationship conflict is −0.236, with a 95% confidence interval of [−0.069, −0.015], which does not include 0. The positive indirect effect of power disparity on team performance through task conflict is 0.104, with a 95% confidence interval of [0.022, 0.081], which also does not include 0. Therefore, H_1_ and H_2_ are further supported, and the results suggest that the negative effect of relationship conflict is greater than the positive effect of task conflict.

#### 4.4.3 The moderating role of power congruence

**Figure 2:**
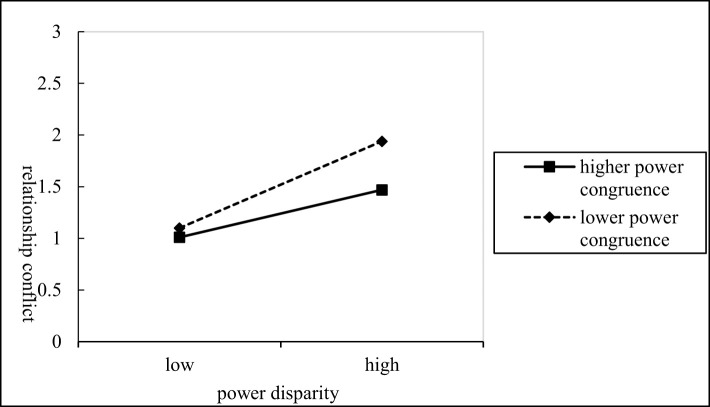
The moderating effect of power congruence in power disparity and relationship conflict.

**Figure 3:**
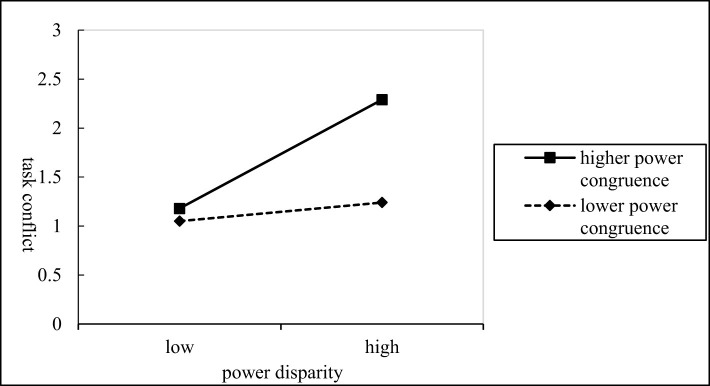
The moderating effect of power congruence in power disparity and task conflict.

From M_6_, it can be inferred that the interaction term (power disparity*power congruence) can affect relationship conflict significantly (β = –0.167*, P* < 0.01), indicating that power congruence plays a moderating role in the relationship between power disparity and relationship conflict negatively. That is, the higher the level of power congruence, the weaker the positive effect of power disparity on relationship conflict. Conversely, the lower the level of power congruence, the stronger the positive effect of power disparity on relationship conflict.The specific moderating effects can be seen in Figure 2, thus verifying H_3_. Meanwhile, from M_9_, it can be inferred that the interaction item can affect task conflict significantly (β = 0.135, *P* < 0.05), thus indicating that power congruence plays a moderating role in the relationship between power disparity and task conflict. That is, the higher the level of power congruence, the stronger the positive effect of power disparity on task conflict. Conversely, the lower the level of power congruence, the weaker the positive effect of power disparity on task conflict.The specific moderating effects can be seen in Figure 3, thus verifying H_4_.

#### 4.4.4 The moderated mediation effect

First, for the moderating effect on the mediator of relationship conflict. The results of the test using 5,000 bootstrapping samples indicate that the 95% CI was (0.016, 0.032) (see table 5), excluding 0, indicating a significant moderated mediation effect. Using Monte Carlo method for further verification, it can be found that the 95% CI of the moderating effect of power congruence on the mediator of relationship conflict is (0.021, 0.078), excluding 0, which also confirmed that the moderating effect on the mediator of relationship conflict is significant, thus verifying H_5_. Second, for the moderating effect on the mediator of task conflict. The results of the test using 5,000 bootstrapping samples indicate that the 95% CI was (0.011, 0.053) (see table 6), excluding 0, indicating a significant moderated mediation effect. Using Monte Carlo method for further verification, it can be found that the 95% CI of the moderating effect of power congruence on the mediator of task conflict is (0.060, 0.149), excluding 0, which also confirmed that the moderating effect on the mediator of task conflict is significant, thus verifying H_6_.

**Table 5:** The results of the moderating effect on the mediator of relationship conflict.

	**EV**	**SE**	**95% CI**
Higher power congruence	0.037	0.024	(0.014, 0.052)
Lower power congruence	0.030	0.022	(0.018, 0.037)
The moderated mediator	0.033	0.019	(0.016, 0.032)

**Table 6:** The results of the moderating effect on the mediator of task conflict.

	**EV**	**SE**	**95% CI**
Higher power congruence	0.036	0.017	(0.007, 0.049)
Lower power l congruence	0.040	0.020	(0.018, 0.061)
The moderated mediator	0.045	0.024	(0.011, 0.053)

## 5 Discussion and implications

### 5.1 Discussion

First, relationship conflict and task conflict both act as mediators in this study. power disparity intensifies relationship conflict within the team by triggering competition among team members and generating negative emotions such as unfair perceptions on the part of team members, thus damaging team performance. It can be seen that relationship conflict plays a mediating role in the relationship between power disparity and team performance. Meanwhile, power disparity also stimulates task conflict and ultimately enhances team performance by causing low-power members to question task goals, priorities, and execution methods. Thus, task conflict plays a partial mediating role in the relationship between power disparity and team performance.

Second, power congruence effectively exerts its moderating effect in this study. It has been confirmed that the moderating effect of power congruence is conveyed by the dual paths of task conflict and relationship conflict, such that the higher power congruence is, the weaker the negative effect of power disparity on team performance. On the one hand, in cases of high power congruence, team members have highly consistent views of the power distribution structure, and they are clearer regarding their position on the team; the fewer instances of overstepping the power boundary occur, the lower the level of relationship conflict caused by power disparity, thus ultimately improving team performance. Simultaneously, in cases of high power congruence, team stability is enhanced, and team members also form confidence expectations, which can effectively reduce the uncertainty associated with team actions, provide a common internal script to promote mutual assistance and coordination, enhance good communication, promote information sharing, improve task conflict, and benefit the team.

### 5.2 Theoretical implications

First, by revealing the negative mechanism underlying the effect of power disparity on team performance based on the power conflict theory, this study enriches the research context of power disparity. In line with power functionalism, the power conflict theory is key theory to explain the effect of power disparity (Greer, 2014). However, literatures on power disparity according to the power conflict theory remains lacking. Accordingly, this study constructed a theoretical model of the effect of power disparity on team performance by identifing team conflict as the mediator and power congruence as the moderator, and confirmed that team conflict is the key process explanatory variable; furthermore, this study found that this negative effect is alleviated under conditions of power congruence. Simultaneously, unlike previous studies, this study distinguished and explored the effect of different types of conflict, thus providing new research ideas that can be used to explore the effect of different types of conflict in further detail.

Second, this study expanded the utility boundary of power disparity. Hitherto, the moderating factors of power disparity have mainly focused on team structure, task type, leadership characteristics and other factors (Xie et al., 2020); however, the influence of power itself has rarely been mentioned. In fact, power itself is believed to be able to affects the utility of power disparity and team processes (Greer, 2014). The factors associated with power itself include power cognition, which mainly includes power congruence and legitimacy (Lammers et al., 2008). Accordingly, this study, from the perspective of the mechanism on which power itself has an impact, identified power congruence as a moderator to verify the changes that occur in team conflict and team performance in cases of power disparity.

### 5.3 Practical implications

First, we distinguished among and utilized different types of conflict. Under previous versions of power conflict theory, team conflict was considered a single overall variable and was considered to affect team performance negatively (Greer et al., 2017). However, the classic literature divided conflict into task and relationship conflicts (Jehn, 1995) and highlighted the fact that they have different effects on team performance (Zhu et al., 2019). This study also proved that realtionship conflict has a negtaive effect on team performance, unlike task conflict. Therefore, this paper makes the following suggestions for enterprises: first, they can avoid and prevent the intensification of relationship conflict by establishing harmonious relationships within the team and implementing a dispute settlement mechanism, and second, they can stimulate task conflict by establishing a regular communication and sharing mechanism and permitting the existence of different opinions.

Second, we should focus on the role of power congruence in this context. People's inconsistent views on power may cause serious damage to team performance (Anderson et al., 2008; Greer, 2014). It is important to improve the team's level of power congruence and to ensure that each member's view of the power level is consistent with the views of other members. This study also conducted empirical research to prove that power congruence improves team conflict and ultimately improves team performance. Enterprises can improve their employees' cognition of the rationality of the hierarchy by making the system design and migration norms of the hierarchy public because once the hierarchy is considered unreasonable, power incongruence is likely to occur (Lammers et al., 2008). Enterprises can also offer team management solutions for work design involving nonoverlapping responsibilities and power bases among team members (to reduce competition on the same power basis) or promote consistent power cognition within the team by providing team training.

### 5.4 Limitations and future directions

First, this study used only aggregate data collected from 62 teams and thus lacked sufficient sample data; accordingly, the research conclusions may exhibit certain deviations. Therefore, in future research, the number of research companies and teams should be increased, the sample size should be expanded, and the research reliability should be improved. Second, by confirming the effective moderating role of power congruence, this paper extends the study on the boundary conditions of power disparity. However, other potential moderating factors should be given more attention in future research, such as the norms of intrateam cooperation (Greer et al., 2011), should be considered and combined with the corresponding cultural context to better meet the needs of management practice (Xie et al., 2020). It is particularly worthwhile to conduct similar studies in cultural contexts with lower power distance, so as to more comprehensively reveal the impact mechanisms of power structures across different cultural backgrounds. Third, this study distinguished different types of conflict and confirmed their mediating effects. However, this study divided conflict only into two dimensions, which cannot fully reflect the effects of conflict. Some scholars have claimed that different types of conflict should be distinguished based on a broader perspective and that the mediating effects of these various types should be confirmed. They have further noted that certain types of conflict related to team power, such as status conflict, can be identified in further detail (Greer et al., 2011). Namely, status conflict within a team hierarchy has a real and powerful objective existence (Bendersky and Hays, 2012). Therefore, in future research, different types of team conflict can be distinguished in further detail, including the newly proposed types of interest conflict and behavior conflict (Ma et al., 2017).

## Data Availability

The raw data supporting the conclusions of this article will be made available by the authors, without undue reservation.
